# Neurocognitive dysfunction and brain FDG-PET/CT findings in HIV-infected hemophilia patients and HIV-infected non-hemophilia patients

**DOI:** 10.1371/journal.pone.0230292

**Published:** 2020-03-19

**Authors:** Koubun Imai, Sota Kimura, Yoko Kiryu, Aki Watanabe, Ei Kinai, Shinichi Oka, Yoshimi Kikuchi, Satoshi Kimura, Mikiko Ogata, Misao Takano, Ryogo Minamimoto, Masatoshi Hotta, Kota Yokoyama, Tomoyuki Noguchi, Kensuke Komatsu

**Affiliations:** 1 Department of Psychiatry, Hitachi Medical Education and Research Center, University of Tsukuba Hospital, Hitachi, Ibaraki, Japan; 2 AIDS Clinical Center, National Center for Global Health and Medicine, Shinjuku, Tokyo, Japan; 3 Tokyo Metropolitan Children’s Medical Center, Fuchu, Tokyo, Japan; 4 Department of Laboratory Medicine, Tokyo Medical University Hospital, Shinjuku, Tokyo, Japan; 5 The Center for Education and Research of Infection Prevention and Control, Tokyo Healthcare University, Shinagawa, Tokyo, Japan; 6 Medical Genomics Center, National Center for Global Health and Medicine, Shinjuku, Tokyo, Japan; 7 Department of Radiology, National Center for Global Health and Medicine, Shinjuku, Tokyo, Japan; 8 Department of Radiology, National Hospital Organization Kyushu Medical Center, Fukuoka, Fukuoka, Japan; Spedali Civili of Brescia, University of Brescia, ITALY

## Abstract

This single-institution cross-sectional study aimed to grasp the prevalence and features of neurocognitive dysfunction in HIV-infected hemophilia patients in Japan. We conducted neuropsychological tests and medical examinations in 56 HIV-infected hemophilia patients who received outpatient treatment at the AIDS Clinical Center, National Center for Global Health and Medicine. A total of 388 HIV-infected non-hemophilia patients who received outpatient treatment at the same institution were included as a control group. To investigate sites responsible for neurocognitive dysfunction in HIV-infected hemophilia patients using brain FDG-PET/CT scans, the accumulation of FDG in each brain region was compared. Approximately 50% of HIV-infected hemophilia patients had neurocognitive dysfunction. The prevalence of asymptomatic neurocognitive impairment was high (34%). Neurocognitive dysfunction was associated with educational level in HIV-infected hemophilia patients. In the symptomatic group, hemophilic arthropathy and history of cerebrovascular disorders were associated with neurocognitive dysfunction. Left temporal lobe function was reduced in the symptomatic group.

## Introduction

HIV-associated neurocognitive disorders (HAND) occur even in patients in whom viral suppression with anti-HIV drugs is favorable [1]. HAND is associated with poor quality of life [2]. According to reviews on neuroimaging studies of HAND, the prevalence of symptomatic HAND is associated with gray matter atrophy in brain magnetic resonance imaging (MRI) [3]. Although a 18-fluorine-fluorodeoxyglucose positron-emission-tomography/computed tomography (^18^F-FDG-PET/CT) scan is not typically recommended for HAND diagnosis [4], FDG-PET has been widely used to investigate cognitive functions and patterns of cognitive alterations [5].

A review of HIV-infected hemophilia outside Japan suggested the presence of both long-term neurocognitive dysfunctions and neurological alterations, such as atrophic changes and lesions in white matter [6]. Another study by the same authors compared hemophilia in HIV-infected and HIV-uninfected patients, and reported that HIV-infected hemophilia patients exhibited deficits in attention, short-term memory, abstraction, and visual recognition [7].

According to an investigation of HAND in Japanese HIV-infected patients, the “Epidemiological study of HIV-associated neurocognitive disorders in Japan” (the J-HAND study), the prevalence of HAND in HIV-infected patients is 25%; asymptomatic neurocognitive impairment (ANI): 13%, mild neurocognitive disorder (MND): 11%, and HIV-associated dementia (HAD): 1% [8]. According to multivariate analysis, age of ≥ 50 years and incomplete virological suppression were identified as risk factors for the symptomatic group, and current treatment with anti-HIV drugs was identified as a protective factor. However, in the J-HAND study, hemophilia patients infected with HIV were excluded. Thus, the presence of cognitive impairments in those patients remains unclear.

The present study is the first report to compare the prevalence of neurocognitive dysfunction in HIV-infected hemophilia patients (NDHH) in Japan and its features with those in HIV-infected non-hemophilia patients, alongside analyzing brain sites involved in NDHH using FDG-PET/CT scanning.

## Methods

### Procedures and participants

This was a cross-sectional observational study approved by the ethics committee of National Center for Global Health and Medicine (“A study of HIV-associated neurocognitive disorders in HIV-infected hemophilia patients”, March 2016, approval number: NCGM-G-001973-0, “A study of neurocognitive dysfunction in HIV-infected hemophilia patients in comparison with HIV-infected non-hemophilia patients and brain FDG-PET/CT findings”, October 2018, approval number: NCGM-G-003055-00). This study was conducted in accordance with the Declaration of Helsinki and its later amendments.

Participants included in the study were HIV-infected hemophilia patients who received outpatient treatment at the AIDS Clinical Center in National Center for Global Health and Medicine (ACC) between May 2016 and February 2018. Exclusion criteria for participants were determined based on the criteria of the Diagnostic and Statistical Manual of Mental Disorders 5th ed. (DSM-5) (9) as follows: (1) those who currently had an active AIDS-defining illness requiring treatment; (2) those with congenital mental retardation; (3) major depressive disorder and/or schizophrenia; (4) Alzheimer’s disease, frontotemporal lobar degeneration, Lewy body dementia, prion diseases, Parkinson’s disease, and/or Huntington’s disease; (5) cerebrovascular disease; (6) traumatic brain lesion; (7) habitual illicit drug users and/or severe alcoholics; (8) those undergoing treatment for central nervous system opportunistic disease or with clear physical impediments; (9) those exhibiting other pathology that clearly caused cognitive impairment; (10) those with fever ≥ 38.5°C or any active infectious symptoms during examination; (11) those in whom neuropsychological testing was judged to be performed inaccurately; (12) those who underwent neuropsychological testing within the past 1 year. Patients with acute or subacute lesions detected on brain MRI that could affect cognitive function and patients with foreign materials that were unacceptable for an MRI scan were also excluded.

When applicable patients visited the hospital for an outpatient visit, the coordinator provided a written explanation of the present study and obtained consent by signature. Thereafter, patient information was collected from medical records. Other necessary information was collected by interviews on the morning of the neuropsychological testing day. Medical examinations were performed by a psychiatrist. In the afternoon, the same neuropsychological tests were performed in one session by the same clinical psychologist as that in the J-HAND study (8).

The following assessment items were collected from medical records and interviews: age, sex, educational level, work experience, presence/absence of persons living with the patients, smoking history, alcohol intake, previous history of AIDS-defining illness, hypertension, diabetes, anemia, dyslipidemia, previous positive history for treponema pallidum hemagglutination test (TPHA), presence of hepatitis B virus/hepatitis C virus (HCV) coinfection, presence/absence of history of HCV treatment, HCV-RNA levels, minimum CD4 count (Nadir CD4), current CD4 count/HIV-RNA levels at the most recent neuropsychological testing, state of introduction of anti-HIV drugs (non-nucleoside reverse transcriptase inhibitor, protease inhibitor, and integrase strand transfer inhibitor), presence/absence of upper-limb dysfunction, classification of coagulation disorders, presence/absence of regular infusion, history of inhibitors, presence/absence of hemophilic arthropathy, history of cerebrovascular disorders, presence/absence of previous virological treatment failure, and presence/absence of previous discontinuation of treatment. Previous virological treatment failure was defined as twice or more viral load > 200 copies/mL of HIV-RNA as tested > 1 month apart at 6 months after start of anti-HIV drugs.

For control group data from HIV-infected non-hemophilia patients, the data obtained at ACC from the J-HAND study was used [8]. Three time zones (9 a.m. to 10 a.m., 10 a.m. to 11 a.m., 11 a.m. and later) were set in each of three outpatient examination rooms. Patients were recruited who met the selection criteria in the order of visit time, and verbal consent was obtained from up to three patients per day in each room. The control group included 388 randomly extracted Japanese HIV-infected patients who received outpatient treatment at the ACC between July 2014 and July 2016 (route of infection: sexual transmission, n = 385; other and unknown, n = 3). Participant exclusion criteria were in accordance with DSM-5 [9].

### Assessment of cognitive functions

Psychiatric symptoms and functional status of daily activities were assessed using the Mini International Neuropsychiatric Interview (M.I.N.I) [10, 11], the Japanese edition of the brief profile of mood states (POMS) [12], the general health questionnaire mental health questionnaire (GHQ-28) [13], and the scale of the 7-item version of the International Classification of Functioning, Disability and Health (ICF) core set [14, 15]. Cognitive functions were assessed with the Mini-Mental State Examination (MMSE) and the following 14 tests in eight cognitive domains [16]: language (language fluency test [category] and [letter]) [17], attention/working memory (recitation in normal order/reverse order) [18, 19], executive functions (Trail Making Test [TMT]-B) [20], visuospatial construction (Rey-Osterreith Complex Figure Test [ROCFT] [copying]) [21, 22], learning (ROCFT [immediate], stories [immediate]) [23], memory (ROCFT [delayed], stories [delayed]), information processing speed (TMT-A, symbol) [18, 19], and motor skills (Grooved Pegboard [GP] [dominant hand] [non-dominant hand]) [24]. Based on the results of age-standardized neuropsychological tests [17, 19, 23–26], patients with scores lower than one or more standard deviations (SDs) were identified. The Frascati Criteria was used for making a diagnosis of neurocognitive dysfunction and determining its severity [27]. On standardized neuropsychological testing, ANI and MND are documented by performance of at least 1SD below the mean involving at least two domains. HAD is ascertained with at least two domains with 2SD or greater than the mean. ANI does not interfere with daily functioning, MND produces at least mild interference, and HAD produces marked interference.

### Assessment of imaging

Brain MRI was performed on a clinical 3.0-Tesla MRI unit (MAGNETOM Verio; Siemens AG, Erlangen, Germany) and included three-dimensional T1-weighted images (magnetization-prepared 180° radio-frequency pulses and rapid gradient-echo sampling, MPRAGE) used for the computed analysis of Voxel-based Specific Regional Analysis System for Alzheimer's Disease (VSRAD) [28], T2*-weighted images used for evaluation of microbleeding, MR angiography used for evaluation of cerebrovascular disorder, diffusion-weighted images used for evaluation of acute or subacute cerebrovascular attack, T2-weighted images, and fluid-attenuated inversion recovery image (FLAIR) used for evaluation of general brain disorders. All MR images were evaluated by an observer (TN) with 10 years of experience in neuroradiology.

For brain FDG-PET/CT scans, ^18^F-FDG was synthesized in the hospital using synthesizing equipment, cyclotron (F200, Sumitomo Heavy Industries, Ltd.) at the National Center for Global Health and Medicine. Participants fasted for over 6 hours. After resting for over 15 minutes with an eye mask in a decubitus position in a dark, quiet room, ^18^F-FDG 5 MBq/kg (lower limit: 185 MBq, upper limit: 370 MBq) was intravenously injected. The condition of wearing an eye mask in a decubitus position in a dark, quiet room was maintained until imaging was performed. Imaging was started with PET/CT equipment (Biograph mCT S20: Siemens Medical Solutions) 45 minutes after ^18^F-FDG administration. Blood glucose levels at scanning were confirmed to be ≤ 200 mg/dL in all participants. The obtained PET data were analyzed using the data analysis software of SIEMENS, MI Neurology, in which a total of 18 regions were set: left/right frontal lobes, left/right temporal lobes, left/right parietal lobes, left/right cingulate and paracingulate gyri, left/right central regions, left/right occipital lobes, left/right basal ganglia, left/right mesial temporal lobes, and left/right cerebellum. The ^18^F-FDG accumulation in each region (SUVmean) was measured, and the SDs of SUVmean were compared. The mean value of the accumulation in the left and right regions whose SD was the smallest was set as the value of the control region. The ratios of the values of the other 16 regions to the control value (SUVr) were calculated for assessment.

### Statistical analysis

For statistical analysis, the statistical software IBM SPSS Statistics ver. 23 was used.

### 1) Assessment of cognitive functions

To examine differences in patient characteristics between HIV-infected hemophilia patients (hemophilia group) and HIV-infected non-hemophilia patients (non-hemophilia group), χ^2^ test or Mann-Whitney U test was performed. To examine differences in the prevalence of neurocognitive dysfunction, cognitive domains, and neuropsychological test results between the groups, the χ^2^ test was performed. Results were considered statistically significant if the P value (p) was < 0.05.

To identify factors associated with NDHH, the χ^2^ test or Mann-Whitney U test was performed between the NDHH group (ANI, MND, and HAD) and normal group (without ANI, MND, or HAD) among HIV-infected hemophilia patients. The χ^2^ test or Mann-Whitney U test was also performed between the symptomatic group (MND and HAD) and asymptomatic group (normal and ANI). Accordingly, factors that were different at a significance level of 5% as explanatory variables were used to perform binary logistic regression. The items from which information could not be obtained were considered missing values.

### 2) Assessment of imaging

The Mann–Whitney U test was performed to compare the NDHH group (ANI, MND, and HAD) and normal group (without ANI, MND, or HAD) as well as the symptomatic group (MND and HAD) and asymptomatic group (normal and ANI) among HIV-infected hemophilia patients. A P value (p) < 0.05 was considered statistically significant.

## Results

In total, 82 HIV-infected hemophilia patients received outpatient treatment at the ACC between May 1, 2016 and February 28, 2018. Of these, eight patients satisfied exclusion criteria, nine patients refused to participate in the study, one patient withdrew consent for participation, and one patient did not undergo neuropsychological testing during the study period; thus, 63 patients underwent neuropsychological testing and medical examination by a psychiatrist. Ten patients were diagnosed with psychiatric disorder in a medical examination by a psychiatrist: schizophrenia (n = 1), bipolar disorder (n = 1), dysthymia (n = 2), developmental disorder (n = 2), alcoholism (n = 1), and sleep disorder (n = 3). Of these, three patients with psychiatric disorders that could affect cognitive function (schizophrenia, bipolar disorder, and alcoholism) and four patients with unavailable recent brain imaging findings were excluded. A final total of 56 patients were included in analysis; however, brain FDG-PET was performed only in 55 patients since 1 patient missed the scheduled examination date.

There were significant differences in most items with regard to patient characteristics between the hemophilia group and non-hemophilia group (Table 1). In the hemophilia group, all patients were male (100%, χ^2^ (1, N = 444) = 3.341, p = 0.048) and had hypertension (43%, χ^2^ (1, N = 444) = 6.375, p = 0.012), diabetes (16%, χ^2^ (1, N = 444) = 9.553, p = 0.006), anemia (16%, χ^2^ (1, N = 444) = 26.084, p < 0.001), HCV antibodies (98%, χ^2^ (1, N = 444) = 322.499, p < 0.001), and previous treatment failure (36%, χ^2^ (1, N = 444) = 24.385, p < 0.001). Additionally, the time after HIV diagnosis (314 [273–332] months, U = 508.000, p < 0.001) and duration of anti-HIV drug therapy (260 [232–290] months, U = 1299.000, p < 0.001) were longer than that in the non-hemophilia group. In contrast, there were fewer patients in the hemophilia group than in the non-hemophilia group with work experience (64%, χ^2^ (1, N = 444) = 9.817, p = 0.002), were living alone (25%, χ^2^ (1, N = 444) = 11.087, p = 0.001), had habitual drinking (34%, χ^2^ (1, N = 444) = 4.073, p = 0.044) or drug use (present) (0%, χ^2^ (1, N = 444) = 3.662, p = 0.036), were previously positive for TPHA (0%, χ^2^ (1, N = 444) = 45.028, p < 0.001), were applicable to the M.I.N.I. items (23%, χ^2^ (1, N = 444) = 10.474, p = 0.001), and had the onset of AIDS (13%, χ^2^ (1, N = 444) = 8.137, p = 0.004).

**Table 1:** Patient characteristics.

	Total n = 444	Hemophilia	Non-hemophilia	p value
		n = 56	n = 388	
Age, median (interquartile range)#	45 (40–53)	47 (43–54)	45 (40–53)	0.065
Sex (male)	422 (95%)	56 (100%)	366 (94%)	0.048
Educational level (university or higher)	213 (48%)	21 (38%)	192 (50%)	0.093
Presence of work experience	355 (80%)	36 (64%)	319 (82%)	0.002
Living alone	203 (46%)	14 (25%)	189 (49%)	0.001
Habitual drinking	206 (47%)	19 (34%)	187 (48%)	0.044
Drug use (present)	24 (5%)	0 (0%)	24 (6%)	0.036
Hypertension (SBP ≧ 140 or DBP ≧ 90 mmHg)	127 (29%)	24 (43%)	103 (27%)	0.012
Diabetes (HbA1c ≧ 7.0 or on treatment)	29 (7%)	9 (16%)	20 (5%)	0.006
Anemia: hemoglobin (M < 12 g/dL, F < 10 g/dL)	17 (4%)	9 (16%)	8 (2%)	< 0.001
HCVAb	71 (16%)	55 (98%)	16 (4%)	< 0.001
Previously positive for TPHA	185 (42%)	0 (0%)	185 (48%)	< 0.001
Applicable to the M.I.N.I. items	192 (43%)	13 (23%)	179 (46%)	0.001
Time after HIV diagnosis (months), median (interquartile range)#	101 (51–173)	314 (273–332)	94 (46–152)	< 0.001
Onset of AIDS	127 (29%)	7 (13%)	120 (31%)	0.004
Current CD4, median (interquartile range)#	531 (390–696)	525 (342–662)	540 (396–700)	0.448
Nadir CD4, median (interquartile range)#	141 (46–228)	141 (91–184)	147 (45–236)	0.433
HIV-RNA<20	400 (90%)	49 (89%)	351 (90%)	0.748
Previous treatment failure	63 (14%)	20 (36%)	43 (11%)	< 0.001
Duration of ART (months), median (interquartile range)#	85 (41–160)	260 (232–290)	78 (36–127)	< 0.001

・Significance test: χ2 test or (#)Mann-Whitney U test

・Significance level: p < 0.05

There were significant differences in most items with regard to patient characteristics between the hemophilia group and non-hemophilia group.

Abbreviations: SBP, systolic blood pressure; DBP, Diastolic blood pressure; HbA1c, hemoglobin A1c; HCVAb, hepatitis C virus antibody; TPHA, treponema pallidum hemagglutination test; M.I.N.I., the Mini International Neuropsychiatric Interview; HIV, human immunodeficiency virus; AIDS, acquired immune deficiency syndrome; RNA, ribonucleic acid; ART, highly active anti-retroviral therapy.

### Comparison of the prevalence of neurocognitive dysfunction in the hemophilia and non-hemophilia groups

**Fig 1:**
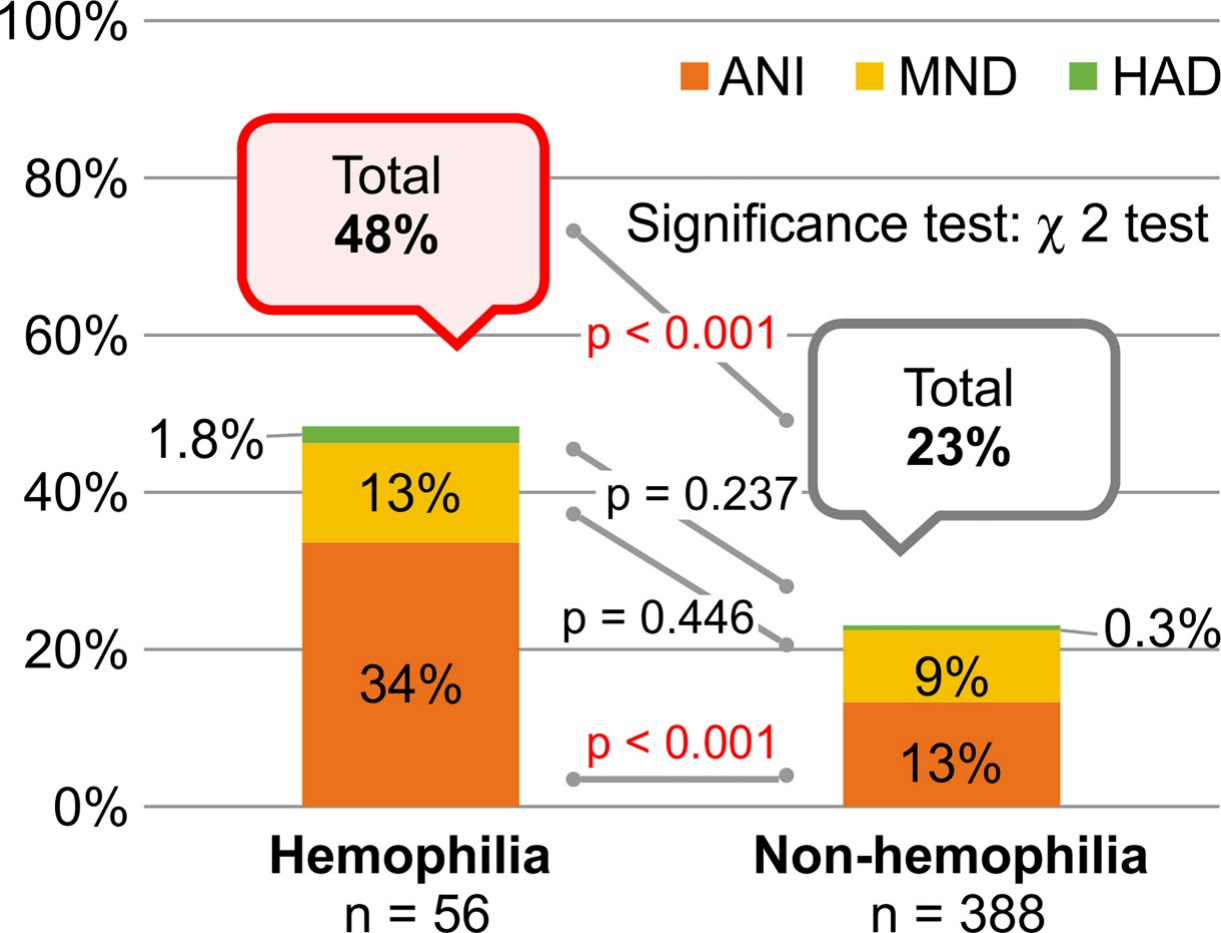
The prevalence of neurocognitive dysfunction in the hemophilia group (in accordance with the Frascati Criteria) of HIV-infected hemophilia patients, 48% had neurocognitive dysfunction that met the diagnostic criteria for HAND. In particular, there was significantly higher prevalence of ANI in the hemophilia group than in the non-hemophilia group. Abbreviations: ANI, asymptomatic neurocognitive impairment; MND, mild neurocognitive disorder; HAD, HIV-associated dementia; HAND, HIV-associated neurocognitive disorders.

Of 56 hemophilia patients, 27 (48%) patients met the diagnostic criteria for HAND using the Frascati Criteria (27): ANI (n = 19, 34%), MND (n = 7, 13%), and HAD (n = 1, 1.8%) (Fig 1). In contrast, of 388 non-hemophilia patients, 89 (23%) patients met the diagnostic criteria for HAND: ANI (n = 52, 13%), MND (n = 36, 9%), and HAD (n = 1, 0.3%). The prevalence of HAND was significantly higher in the hemophilia group than in the non-hemophilia group (χ^2^ (1, N = 444) = 16.199, p < 0.001). In particular, there was significantly higher prevalence of ANI in the hemophilia group than in the non-hemophilia group (χ^2^ (1, N = 444) = 15.348, p < 0.001).

**Fig 2:**
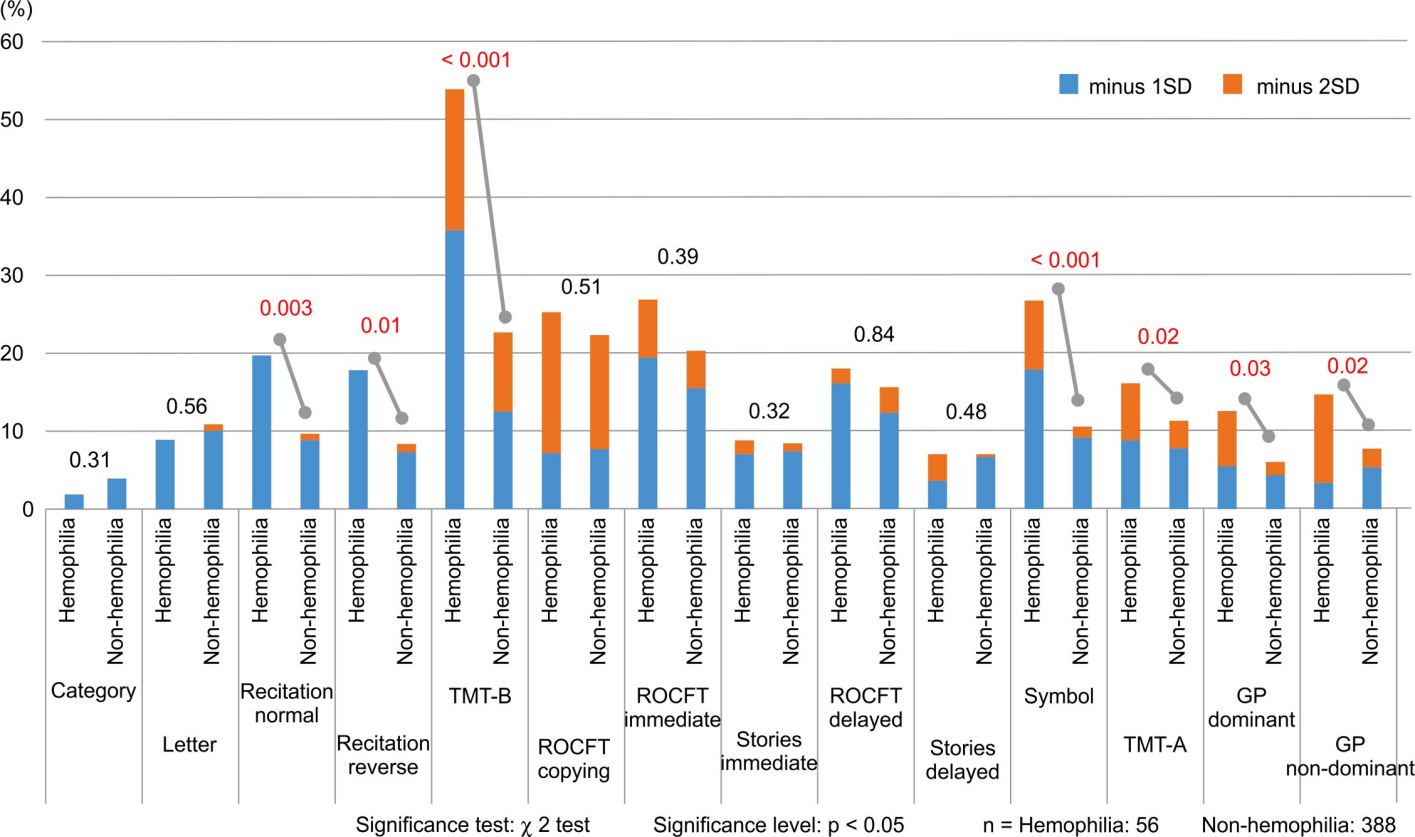
Comparison of the proportions of low scores (at least 1SD below the mean) on psychological testing. A comparison of the proportions of low scores (at least 1SD below the mean on standardized neuropsychological tests) showed that recitation in normal order, recitation in reverse order, TMT-B, symbol, TMT-A, GP (dominant hand), and GP (non-dominant hand) were significantly higher in the hemophilia group than in the non-hemophilia group. Abbreviations: SD, standard deviation; TMT, Trail Making Test; ROCFT, Rey-Osterreith Complex Figure Test; GP, Grooved Pegboard.

A comparison of the proportions of low scores (at least 1SD below the mean) on standardized neuropsychological tests [17, 19, 23–26] showed that recitation in normal order (20%, χ^2^ (1, N = 444) = 10.730, p = 0.003), recitation in reverse order (18%, χ^2^ (1, N = 444) = 8.175, p = 0.01), TMT-B (54%, χ^2^ (1, N = 444) = 33.906, p < 0.001), symbol (27%, χ^2^ (1, N = 444) = 16.961, p < 0.001), TMT-A (16%, χ^2^ (1, N = 444) = 6.415, p = 0.02), GP (dominant hand) (12%, χ^2^ (1, N = 438) = 5.504, p = 0.03), and GP (non-dominant hand) (15%, χ^2^ (1, N = 438) = 6.101, p = 0.03) were significantly higher in the hemophilia group than in the non-hemophilia group (Fig 2). A comparison of the proportions of low scores by cognitive domain showed that attention/working memory (13%, χ^2^ (1, N = 444) = 8.587, p = 0.009), executive functions (54%, χ^2^ (1, N = 444) = 33.906, p < 0.001), information processing speed (23%, χ^2^ (1, N = 444) = 14.156, p < 0.001), and motor skills (16%, χ^2^ (1, N = 438) = 9.275, p = 0.006) were significantly higher in the hemophilia group than in the non-hemophilia group. Analysis of the sensitivity and specificity of each test for neurocognitive dysfunction showed a high sensitivity of TMT-B (92.6%) (Table 2).

**Table 2:** The sensitivity and specificity for the NDHH group in each test.

**Category**	Dysfunction	Normal	Total	**Letter**	Dysfunction	Normal	Total	**Recitation in normal order**	Dysfunction	Normal	Total
≦−1SD	1	0	1	≦−1SD	3	2	5	≦−1SD	10	1	11
Normal	26	29	55	Normal	24	27	51	Normal	17	28	45
Total	27	29	56	Total	27	29	56	Total	27	29	56
Sensitivity:	3.7%			Sensitivity:	11.1%			Sensitivity:	37.0%		
Specificity:	100.0%			Specificity:	93.1%			Specificity:	96.6%		
**Recitation in reverse order**	Dysfunction	Normal	Total	**Symbol**	Dysfunction	Normal	Total	**TMT-A**	Dysfunction	Normal	Total
≦−1SD	9	1	10	≦−1SD	13	2	15	≦−1SD	9	0	9
Normal	18	28	46	Normal	14	27	41	Normal	18	29	47
Total	27	29	56	Total	27	29	56	Total	27	29	56
Sensitivity:	33.3%			Sensitivity:	48.1%			Sensitivity:	33.3%		
Specificity:	96.6%			Specificity:	93.1%			Specificity:	100.0%		
**TMT-B**	Dysfunction	Normal	Total	**Rey (copying)**	Dysfunction	Normal	Total	**Rey (immediate)**	Dysfunction	Normal	Total
≦−1SD	25	5	30	≦−1SD	12	2	14	≦−1SD	10	5	15
Normal	2	24	26	Normal	15	27	42	Normal	17	24	41
Total	27	29	56	Total	27	29	56	Total	27	29	56
Sensitivity:	92.6%			Sensitivity:	44.4%			Sensitivity:	37.0%		
Specificity:	82.8%			Specificity:	93.1%			Specificity:	82.8%		
**Rey (delayed)**	Dysfunction	Normal	Total	**Stories (immediate)**	Dysfunction	Normal	Total	**Stories (delayed)**	Dysfunction	Normal	Total
≦−1SD	9	1	10	≦−1SD	5	0	5	≦−1SD	4	0	4
Normal	18	28	46	Normal	22	29	51	Normal	23	29	52
Total	27	29	56	Total	27	29	56	Total	27	29	56
Sensitivity:	33.3%			Sensitivity:	18.5%			Sensitivity:	14.8%		
Specificity:	96.6%			Specificity:	100.0%			Specificity:	100.0%		
**GP (dominant)**	Dysfunction	Normal	Total	**GP (non-dominant)**	Dysfunction	Normal	Total				
≦−1SD	5	2	7	≦−1SD	6	2	8				
Normal	22	27	49	Normal	21	27	48				
Total	27	29	56	Total	27	29	56				
Sensitivity:	18.5%			Sensitivity:	22.2%						
Specificity:	93.1%			Specificity:	93.1%						

*Numbers represent the number of patients

Analysis of the sensitivity and specificity of each test for neurocognitive dysfunction showed a high sensitivity of TMT-B.

Abbreviations: NDHH, neurocognitive dysfunction in HIV-infected hemophilia; SD, standard deviation; TMT, Trail Making Test; GP, Grooved Pegboard.

### Associated factors of NDHH

The results of the χ^2^ test or Mann-Whitney U test for the analysis of associated factors in the NDHH group between the NDHH group and normal group are shown in Table 3. A binary logistic regression was performed using educational level (university or higher) (p = 0.023), and the presence of history of cerebrovascular disorder (p = 0.045), which were significantly different at a significance level of 5% as explanatory variables. This suggested that educational level (university or higher) (odds ratio [OR], 0.267; 95% confidence interval [CI] 0.083–0.854, p = 0.026) was an associated protective factor in the NDHH group (χ^2^ test, p = 0.021, Nagelkerke R^2^, 0.121; sensitivity, 77.8%; specificity, 51.7%).

**Table 3:** Associated factors in the NDHH group.

	Total n = 56	Normal n = 29	NDHH (ANI・MND・HAD) n = 27	χ^2^ value or U value	p value	Multivariate analysis	
						Odds ratio (95% CI)	p value
Hemophilia A	45 (80%)	22 (76%)	23 (85%)	0.770	0.380		
B	11 (20%)	7 (24%)	4 (15%)				
***Educational level (university or higher)***	***21 (38%)***	***15 (52%)***	***6 (22%)***	***5*.*192***	***0*.*023***	***0*.*267 (0*.*083–0*.*854)***	***0*.*026***
Presence of work experience	36 (64%)	21 (58%)	15 (42%)	1.731	0.188		
Presence of smoking history	30 (54%)	12 (41%)	18 (67%)	3.595	0.058		
Anemia:hemoglobin (<12 g/dl)	9 (16%)	2 (7%)	7 (26%)	3.754	0.057		
Presence of upper-limb dysfunction	23 (41%)	13 (45%)	10 (37%)	0.351	0.554		
Presence of hemophilic arthropathy	30 (54%)	16 (55%)	14 (52%)	0.062	0.803		
***Presence of history of cerebrovascular disorder***	***14 (25%)***	***4 (14%)***	***10 (37%)***	***4*.*029***	***0*.*045***		
Current CD4, median (interquartile range)#	525 (342–662)	552 (429–664)	479 (279–684)	303.5	0.215		
Nadir CD4, median (interquartile range)#	141 (91–184)	149 (31–221)	137 (100–161)	342.5	0.561		
Previous treatment failure	20 (36%)	11 (38%)	9 (33%)	0.129	0.720		

・Significance test: χ2 test or (#)Mann-Whitney U test

・Significance level: p < 0.05

χ^2^ test: p = 0.021

Nagelkerke R^2 =^ 0.121

Classification of cases (sensitivity) 77.8%; non-cases (specificity) 51.7%

Binary logistic regression revealed that educational level (university or higher), and the presence of history of cerebrovascular disorder, which were significantly different at a significance level of 5%, were used as explanatory variables. This suggested that educational level was an associated protective factor in the NDHH group.

Abbreviations: NDHH, neurocognitive dysfunction in HIV-infected hemophilia patients; ANI, asymptomatic neurocognitive impairment; MND, mild neurocognitive disorder; HAD, HIV-associated dementia; CI: confidence interval.

We performed the χ^2^ test or Mann-Whitney U test to analyze factors associated with symptoms of neurocognitive dysfunction between the symptomatic and asymptomatic groups (Table 4). Binary logistic regression revealed that the presence of smoking history (p = 0.041), hemophilic arthropathy (p = 0.041), and history of cerebrovascular disorder (p = 0.018), which were significantly different at a significance level of 5%, were used as explanatory variables. The presence of hemophilic arthropathy (OR, 11.998; 95% CI, 1.130–127.403; p = 0.039) and history of cerebrovascular disorder (OR, 10.993; 95% CI, 1.779–67.922; p = 0.010) were suggested to be associated risk factors in the group with symptoms of neurocognitive dysfunction (χ^2^ test, p = 0.002; Nagelkerke R^2^, 0.352; sensitivity, 62.5%; specificity, 95.8%).

**Table 4:** Associated factors in the symptomatic group.

	Total n = 56	asymptomatic (Normal・ANI) n = 48	symptomatic (MND・HAD) n = 8	χ^2^ value or U value	p value	Multivariate analysis	
						Odds ratio (95% CI)	p value
Hemophilia A	45 (80%)	39 (81%)	6 (75%)	0.170	0.497		
B	11 (20%)	9 (19%)	2 (25%)				
Educational level (university or higher)	21 (38%)	20 (42%)	1 (13%)	2.489	0.116		
Presence of work experience	36 (64%)	33 (69%)	3 (38%)	2.917	0.097		
***Presence of smoking history***	***30 (54%)***	***23 (48%)***	***7 (88%)***	***4*.*320***	***0*.*041***		
Anemia:hemoglobin (<12 g/dl)	9 (16%)	6 (67%)	3(33%)	3.177	0.108		
Presence of upper-limb dysfunction	23 (41%)	19 (40%)	4 (50%)	0.307	0.428		
***Presence of hemophilic arthropathy***	***30 (54%)***	***23 (48%)***	***7 (88%)***	***4*.*320***	***0*.*041***	***11*.*998 (1*.*130–127*.*403)***	***0*.*039***
***Presence of history of cerebrovascular disorder***	***14 (25%)***	***9 (19%)***	***5 (63%)***	***7*.*000***	***0*.*018***	***10*.*993 (1*.*779–67*.*922)***	***0*.*010***
Current CD4, median (interquartile range)#	525 (342–662)	525 (361–645)	289 (229–784)	175.0	0.770		
Nadir CD4, median (interquartile range)#	141 (91–184)	141 (91–175)	132 (75–186)	161.0	0.872		
Previous treatment failure	20 (36%)	18 (38%)	2 (25%)	0.467	0.399		

・Significance test: χ2 test or (#)Mann-Whitney U test

・Significance level: p < 0.05

χ^2^ test; p = 0.002

Nagelkerke R^2^ = 0.352

Classification of cases (sensitivity) 62.5%; non-cases (specificity) 95.8%

Binary logistic regression revealed that the presence of smoking history, hemophilic arthropathy, and history of cerebrovascular disorder, which were significantly different at a significance level of 5%, were used as explanatory variables. The presence of hemophilic arthropathy and history of cerebrovascular disorder were suggested to be associated risk factors in the group with symptoms of neurocognitive dysfunction.

Abbreviations: ANI, asymptomatic neurocognitive impairment; MND, mild neurocognitive disorder; HAD, HIV-associated dementia; CI: confidence interval.

### Brain FDG-PET/CT findings in HIV-infected hemophilia patients

One patient who missed the FDG-PET examination date fell under MND. Therefore, of the 55 patients analyzed, 29 were in normal, 19 in ANI, 6 in MND, and 1 in HAD group. The mean value of SUVmean in the left cerebellum (4.70 ± 0.91) and the right cerebellum (4.88 ± 0.95) was 4.79 ± 0.93, and these SDs were the smallest. There was no difference in FDG accumulation between the NDHH group and normal group. Compared to the asymptomatic group, the symptomatic group exhibited a significant decrease in accumulation in the left temporal lobe (p = 0.021) (Table 5).

**Table 5:** Comparison of SUVr values between the normal group and the NDHH group and between asymptomatic and symptomatic groups among HIV-infected hemophilia patients.

	Normal n = 29	NDHH (ANI・MND・HAD) n = 26	p value	asymptomatic (Normal・ANI) n = 48	symptomatic (MND・HAD) n = 7	p value
Frontal lobe (L)	1.19	1.16	0.187	1.18	1.12	0.142
Frontal lobe (R)	1.19	1.16	0.182	1.18	1.15	0.321
***Temporal lobe (L)***	1.16	1.12	0.063	***1*.*15***	***1*.*06***	***0*.*021***
Temporal lobe (R)	1.19	1.17	0.435	1.19	1.15	0.479
Parietal lobe (L)	1.17	1.13	0.218	1.16	1.09	0.193
Parietal lobe (R)	1.18	1.15	0.367	1.17	1.14	0.520
Cingulate and paracingulate gyri (L)	1.19	1.17	0.386	1.19	1.14	0.279
Cingulate and paracingulate gyri (R)	1.23	1.20	0.395	1.22	1.18	0.341
Central region (L)	1.12	1.10	0.238	1.11	1.08	0.354
Central region (R)	1.15	1.11	0.358	1.14	1.09	0.291
Occipital lobe (L)	1.19	1.15	0.155	1.18	1.14	0.268
Occipital lobe (R)	1.21	1.20	0.395	1.21	1.20	0.662
Basal ganglia (L)	1.23	1.20	0.225	1.22	1.16	0.133
Basal ganglia (R)	1.20	1.18	0.386	1.19	1.18	0.135
Mesial temporal lobe (L)	0.92	0.91	0.652	0.92	0.88	0.105
Mesial temporal lobe (R)	0.93	0.93	0.795	0.93	0.92	0.662

・The SDs of the accumulation in the brain parenchyma (SUVmean) were compared, and the mean value of the accumulation in the left and right regions of the cerebellum, whose SD was the smallest, was used as the value of the control region.

・Significance test: Mann–Whitney U test

・Significance level: p < 0.05

There was no difference in 18-fluorine-fluorodeoxyglucose accumulation between the NDHH group and normal group. Compared to the asymptomatic group, the symptomatic group exhibited a significant decrease in accumulation in the left temporal lobe.

Abbreviations: SUV, standardized uptake value; NDHH, neurocognitive dysfunction in HIV-infected hemophilia patients; ANI, asymptomatic neurocognitive impairment; MND, mild neurocognitive disorder; HAD, HIV-associated dementia; SD, standard deviation; L, left; R, right.

## Discussion

The present study suggested that the prevalence of NDHH was higher than that of HIV-infected non-hemophilia patients. In particular, the prevalence of ANI which does not hamper daily activities was higher. At present, the level of NDHH is mild in most patients, but cerebrovascular aging eventually leads to cognitive decline [29], so it may gradually advance as patients become older. Intracerebral hemorrhage is a known factor that increases the risk of developing cognitive impairment [29]. In the symptomatic group, since hemophilic arthropathy and presence of a history of cerebrovascular disorder were identified as risk factors for neurocognitive dysfunction, lifestyle review and regular administration of blood products are also important, and medical staff/supporters are required to manage these patients. In contrast, educational level (university or higher) was an associated protective factor in the NDHH group in this study, and it has been reported that higher education has a protective effect against neurocognitive impairments even for HIV-infected patients [30]. In the future, nurturing professionals who can provide support for these associated factors as rapidly as possible and prepare an appropriate medical environment for neurocognitive dysfunction are required.

TMT tasks are reported to be very sensitive to differences between a mild Alzheimer’s disease group and a control group [31]. In clinical settings, where the assessment of cognitive functions in eight cognitive domains (verbal/language, attention/working memory, abstraction/executive, memory, speed of information processing, sensory-perceptual, and motor skills) [27] is difficult due to the use of multiple neuropsychological tests (lack of manpower, limited time for medical examination, etc.), conducting TMT-B may be useful as an easy assessment given its high sensitivity/specificity for NDHH. Although the brain sites responsible were not clarified in the NDHH group, the present study suggested that left temporal lobe function was reduced in the symptomatic group. Reduced FDG-PET metabolism within the temporo-parietal and prefrontal cortex were reported in elderly healthy control subjects that developed mild cognitive impairment or Alzheimer’s disease, and the combination of TMT-B and FDG-PET/CT are considered useful for prediction of the clinical progress to dementia [32]. Although there have been studies indicating a significant relationship between temporal lobe metabolism and the severity of AIDS dementia [33], the decreasing pattern of FDG uptake in patients with HAND was considered nonspecific [4]. Of 56 hemophilia patients, 14 (25%) patients had history of cerebrovascular disorder. However, we were unable to identify the relationship between sites and hypometabolism because some patients lack medical records of the site, and some sites were unclear in current MRI.

In studies comparing HIV-infected hemophilia patients and HIV-uninfected hemophilia patients, HIV-infected hemophilia patients were associated with a decline in neurocognitive performance [6, 7]. In our study comparing HIV-infected hemophilia patients and HIV-infected non-hemophilia patients, the prevalence of NDHH was high, and attention was impaired. These results suggest that both HIV infection and hemophilia may be risk factors for neurocognitive dysfunction. In the future, it will be necessary to conduct a prospective cohort study while observing whether subjects develop dementia in combination with brain MRI and molecular imaging modalities.

This study has several limitations. Many participants lacked records of coagulation factor activity during examination. Similarly, accurate analysis of the age of onset of intracranial hemorrhage, site, and physical impediment was difficult, as past medical records did not exist and confirmation was unavailable. For hemophilic arthropathy, we referred to medical records by physicians and did not base diagnosis on evaluation standards such as the hemophilia joint health score (HJHS) or Pettersson score [34, 35]. Since upper-limb dysfunction was judged by patients in interviews and not by disabilities of the arm, shoulder, and hand (DASH) [36], judgments may have varied depending on patients’ subjectivity. Thus, accurate assessments are required using objective data with regard to hemophilia-related items for the analysis of associated factors in the NDHH. In the present study, we assessed TMT and GP using international standard values as standardized Japanese data were not available, and we did not use tests standardized for education level. At present, neuropsychological tests standardized for sex and education level are limited in Japan; thus, advancement in the neuropsychological field is desirable. In addition, the J-HAND study demonstrated that age ≥ 50 years and incomplete virological suppression were identified as risk factors for the symptomatic group, and current treatment with anti-HIV drugs was identified as a protective factor for neurocognitive dysfunction [8]. However, the present participants were a homogeneous population with regard to these factors. Comparison with HIV-uninfected hemophilia patients in Japan to analyze causes of neurocognitive dysfunction are required in the future.

## Conclusion

In the present study, 48% of HIV-infected hemophilia patients had neurocognitive dysfunction that met the diagnostic criteria for HAND. In particular, the prevalence of ANI was high. The analysis of associated factors with neurocognitive dysfunction identified educational level in the NDHH group and hemophilic arthropathy and history of cerebrovascular disorder in the symptomatic group. TMT-B may be useful for the rapid screening of neurocognitive dysfunction in busy medical facilities. In addition, the present study suggested that left temporal lobe function was reduced in the symptomatic group. Thus, longitudinal follow-up of the asymptomatic group by brain FDG-PET/CT may improve understanding of the prognosis of neurocognitive dysfunction. In the future, for HIV-infected hemophilia patients to easily receive treatment and support when they manifest symptoms of neurocognitive dysfunction, professionals with the requisite knowledge and skills to appropriately assess and support patients are necessary, alongside a medical environment that promotes patient support and prevention of the initial onset and progression of neurocognitive dysfunction.

## Supporting information

S1 Dataset (XLSX)
